# P-2150. Eosinophilia in Patients with Suspected Parasitic Infection: a Retrospective Analysis

**DOI:** 10.1093/ofid/ofae631.2304

**Published:** 2025-01-29

**Authors:** Evgenii Filippov, Jessica Bass, Jiana Blaha, Elise M O’Connell, Amy KLion

**Affiliations:** Sinai Hospital of Baltimore, Baltimore, Maryland; National Institutes of Health, Bethesda, Maryland; National Institutes of Health, Bethesda, Maryland; National Institute of Allergy and Infectious Diseases, Bethesda, Maryland; National Institutes of Health, Bethesda, Maryland

## Abstract

**Background:**

Eosinophilia, defined as an absolute eosinophil count (AEC) ≥500 cells/μL, is common in the setting of helminth infection, though the relationship between the degree of eosinophilia and the likelihood of an underlying helminth infection remains unstudied. To begin to address this question, we performed a retrospective analysis of data from a large cohort of patients referred to the National Institutes of Health for parasitic disease screening.

**Methods:**

Patients evaluated at the NIH from September 2001 to March 2021 on clinical protocols designed to study suspected parasitic infections (NCT00001230 and NCT00001645) and for whom clinical and laboratory information was available in a research database were included. Demographic information, clinical manifestations, final diagnosis, and laboratory values were reviewed. Peak AEC was recorded and categorized as mild (500-1499 cells/μL), moderate (1500-4999 cells/μL), or severe (>5000 cells/μL).

**Results:**

A total of 196 of the 459 included participants (43%) had documented eosinophilia, of whom 61% (n=120), 34% (n=66) and 5% (n=10) had mild, moderate, or severe peak AEC elevations, respectively. Most of the eosinophilic patients were diagnosed with one or more helminth infections (n=150). The remaining patients (n=46) were found to have myiasis (n=1), a non-infectious or no cause identified (n=43) or were lost to follow up (n=2). Among the helminth-infected patients, GM AEC was 1412 cells/μL (range 500-12,800). Most patients likely acquired infection in Africa (n=88) or Central America (n=25). Three patients had no history of travel outside the US. Strongyloidiasis (n=64) and loiasis (n=43) were the most common helminth diagnoses, and 29 individuals had multiple helminths identified. Of the 10 patients in the total patient cohort with peak AEC >5000 cells/μL, 5 were diagnosed with helminth infection (loiasis or strongyloidiasis), none of whom had AEC >20,000 cells/μL.

**Conclusion:**

The study underscores that helminths, particularly strongyloidiasis and loiasis, are commonly associated with mild to moderate eosinophilia. Severe eosinophilia (AECs >5000 cells/μL) is less common and extremely high counts (AEC ≥20,000 cell/μL) should prompt investigation of non-helminth etiologies.

**Disclosures:**

All Authors: No reported disclosures

